# Exosomes: A Forthcoming Era of Breast Cancer Therapeutics

**DOI:** 10.3390/cancers13184672

**Published:** 2021-09-17

**Authors:** Banashree Bondhopadhyay, Sandeep Sisodiya, Faisal Abdulrahman Alzahrani, Muhammed A. Bakhrebah, Atul Chikara, Vishakha Kasherwal, Asiya Khan, Jyoti Rani, Sajad Ahmad Dar, Naseem Akhter, Pranay Tanwar, Usha Agrawal, Showket Hussain

**Affiliations:** 1 ICMR-National Institute of Cancer Prevention and Research, Noida 201301, India; bbanerjee218@gmail.com (B.B.); sandeepsisodiya99@gmail.com (S.S.); atul.chikara5@gmail.com (A.C.); vishakha.kasherwal@s.amity.edu (V.K.); jyotiranibio2018@gmail.com (J.R.); 2 Symbiosis School of Biological Sciences, Symbiosis International (Deemed University), Pune 411004, India; 3 Department of Biochemistry, Faculty of Science, Embryonic Stem Cells Unit, King Fahd Medical Research Center, King Abdulaziz University, Jeddah 21589, Saudi Arabia; faahalzahrani@kau.edu.sa; 4 Life Science and Environment Research Institute, King Abdulaziz City for Science and Technology (KACST), Riyadh 11442, Saudi Arabia; mbakhrbh@kacst.edu.sa; 5 Amity Institute of Molecular Medicine and Stem Cell Research, Amity University, Noida 201313, India; 6 Centre for Medical Biotechnology, Amity Institute of Biotechnology, Amity University, Noida 201313, India; asiya.khan@student.amity.edu; 7 Laboratory Oncology Unit, Dr. Bheem Rao Ambedkar Institute Rotary Cancer Hospital (Dr. BRA-IRCH), All India Institute of Medical Sciences, Ansari Nagar, New Delhi 110023, India; pranaytanwar@aiims.edu; 8 Research and Scientific Studies Unit, College of Nursing, Jazan University, Jazan 45142, Saudi Arabia; sdar@jazanu.edu.sa; 9 Department of Laboratory Medicine, Faculty of Applied Medical Sciences, Albaha University, Albaha 65411, Saudi Arabia; nakhter@bu.edu.sa; 10 ICMR-National Institute of Pathology, New Delhi 110029, India; director-nip@icmr.gov.in

**Keywords:** exosomes, small extracellular vesicles, breast cancer, cancer aggressiveness, multi-drug resistance, diagnosis, immune response, immunotherapy

## Abstract

**Simple Summary:**

Breast cancer prevalence is a major challenge worldwide due to the lack of early diagnostics and treatment modalities. In this era of technological advancements, researchers are exploring several grey areas in breast cancer research, which may lead to the appropriate point of care, non-invasive and diagnostic aid for early breast cancer detection and management. Exosome-based research, an emerging area, endeavors to locate and elucidate the role of exosomes in breast cancer diagnostics, immune response and clinical outcomes. This review may provide insights on small extracellular vesicles research and their role in breast cancer. Future extensive studies on exosome biology in conjunction with cancer genetics shall undoubtedly open up new vistas in exosome-based diagnostics for early cancer detection and therapeutics.

**Abstract:**

Despite the recent advancements in therapeutics and personalized medicine, breast cancer remains one of the most lethal cancers among women. The prognostic and diagnostic aids mainly include assessment of tumor tissues with conventional methods towards better therapeutic strategies. However, current era of gene-based research may influence the treatment outcome particularly as an adjunct to diagnostics by exploring the role of non-invasive liquid biopsies or circulating markers. The characterization of tumor milieu for physiological fluids has been central to identifying the role of exosomes or small extracellular vesicles (sEVs). These exosomes provide necessary communication between tumor cells in the tumor microenvironment (TME). The manipulation of exosomes in TME may provide promising diagnostic/therapeutic strategies, particularly in triple-negative breast cancer patients. This review has described and highlighted the role of exosomes in breast carcinogenesis and how they could be used or targeted by recent immunotherapeutics to achieve promising intervention strategies.

## 1. Introduction

Breast cancer, a heterogeneous disease, is a common cause of death in females worldwide [1,2,3]. The current treatment strategies are based on the expression pattern of the estrogen receptor (ER), the progesterone receptor (PR) and the ERBB2 receptor (Her2) profile [4,5]. Recently, breast cancer survival rate has improved due to outcomes in the primary molecular sub-classification when administered with targeted therapies such as hormone therapy and HER2-targeted therapy (e.g., trastuzumab) [6]. As per the gene expression pattern of breast cancer patients, clustering leads to five different molecular subtypes of breast cancer, i.e., normal type, basal type, Her2-rich, luminal A and luminal B [7], and classifies ER- breast cancer into four different subtypes and triple-negative breast cancers (TNBCs) into six subtypes [8]. The current understanding of breast cancer biology has led to significant improvements in diagnostic and prognostic methods and enhanced novel targeted therapies. However, the limited knowledge about the molecular processes or mechanisms involved in breast cancer pathogenesis has led to restricted therapeutic approaches and poor prognosis of breast cancer patients. Studies have recently elucidated the role of a typical vesicular structure of 30–150 nm diameter called exosomes and/or small extracellular vesicles (sEVs), secreted by various immune cells such as dendritic and Chimeric Antigen Receptor T cells (CAR-T) cells to provide robust diagnostics and therapeutic interventions [9,10]. In the year 1985, exosomes were initially described as a budding membrane of intracellular vesicles [11]. However, recently, stem cells, endothelial cells, dendritic cells, B cells, T cells and especially cancer cells were found to secrete exosomes [12], that can play a crucial role in cell signaling communication, in both paracrine and autocrine manner [13]. Exosomes also assist in transporting various molecules, including proteins, lipids, DNA, mRNA, micro RNAs (miRNA) and lncRNA (Long noncoding RNA) [14,15]. Moreover, exosomes are found amply in pathological and/or physiological fluids, such as breast milk, cerebrospinal fluid, serum, saliva, urine, plasma and ascites [16], making them promising target molecules as cancer cells release more exosomes than non-cancer cells.

## 2. Exosomes: Structure and Functions

Exosomes, first identified by Johnstone et al., are nanovesicles derived from cultured monolayer cells [17], made of growing intracellular endosomes that produce multicellular bodies (MVBs) fused with plasma membranes to secrete exosomes out of the cells [11,18]. Exosomes are lipid vesicles with a bilayer structure and a diameter of 30 to 150 nm [10,19,20], and a buoyant density of 1.13 g/mL to 1.19 g/mL [21]; formed during the process of endosomal maturation by dependent and independent endosomal sorting complexes required for transport (ESCRT) processes [22]. They express several proteins including protein/tetraspanin markers such as TSG101, ALIX, CD63, HSP70, tetraspanin 1–19, Putative tetraspanin-19, Uroplakin-1a,1b, Peripherin-2, CD Antigen 9, 63, 81, 82, 151 and Leucocyte surface antigen CD53, CD37 which play a key role in vesicle detection [23,24,25].

**Figure 1:**
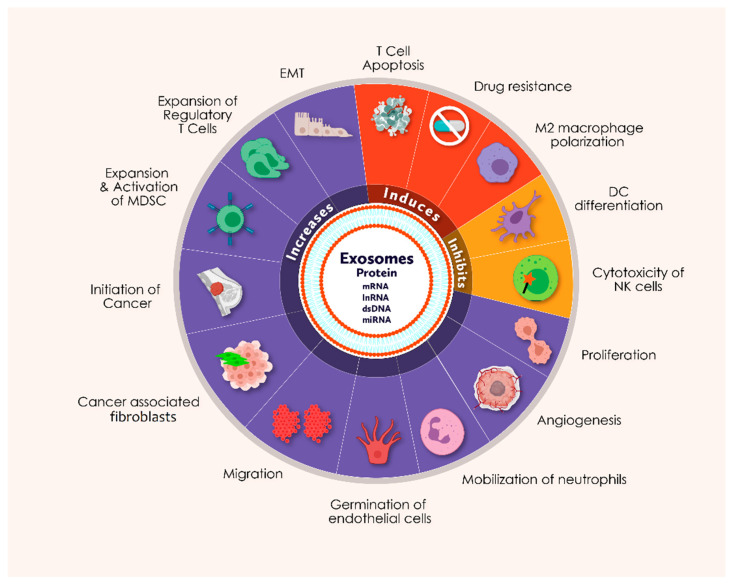
The schematic figure represents the functional abilities of exosomes that may be involved in various cellular processes during breast carcinogenesis (Icons are created with biorender.com (accessed on 20 June 2021)).

Exosomes are a crucial element in the metastasis, development and treatment efficacy of cancer. They also play a key role in tumor development owing to their ten times higher secretion efficiency in cancer cells than in normal cells, resulting in cellular contact in the tumor niche through nucleic acid and oncogenic protein transmission [26,27,28,29,30,31,32,33,34]. The absorption of exosomes induces upregulation of genes related to angiogenesis, leading to proliferation, migration and germination of endothelial cells [35]. In the premetastatic niche, exosomes help in epithelial to mesenchymal transition (EMT) through distant metastasis [36,37,38], and also contribute to cancer-associated fibroblasts (CAFs) for the enhancement of cancer aggressiveness. Exosomes are also involved in neutrophil deployment, growth and stimulation of myeloid-derived suppressor cells (MDSC), inhibition of dendritic cell (DC) differentiation, inhibition of natural killer cells (NK) cytotoxicity, induction of M2 polarization of macrophages, development of regulatory T cells (Treg) and induction of apoptosis of cytotoxic T (Tc) cells [39,40,41,42]. Exosomes not only contribute to the growth of cancer cells but also provide chemoresistance to the neighboring cells in the tumor microenvironment against various chemotherapeutic agents, displaying the role as a safeguard for other cancer cells [43,44]. Various in-vitro studies and clinical studies on breast cancer have demonstrated that exosomes might contribute to miRNA processing delivery and result in induction of tumor formation and/or transformation in non-tumorigenic breast cells [45]. In addition, autocrine signaling has been found to trigger further cancer progression via exosomes derived from the cancer cells. For example, exosomes extracted from in vitro gastric cancer cells encourage growth via Akt/PI3K (Phosphoinositide 3-Kinase), MAPK (Mitogen Activated Protein Kinase) and Notch-1 dependent signaling pathways [46,47]. Overall, cancer cells can customize isomorphic exosomes to guide cancer progression by targeting the different molecules and processes related to breast carcinogenesis (Figure 1).

## 3. Origin-Based Types of Exosomes

**Figure 2:**
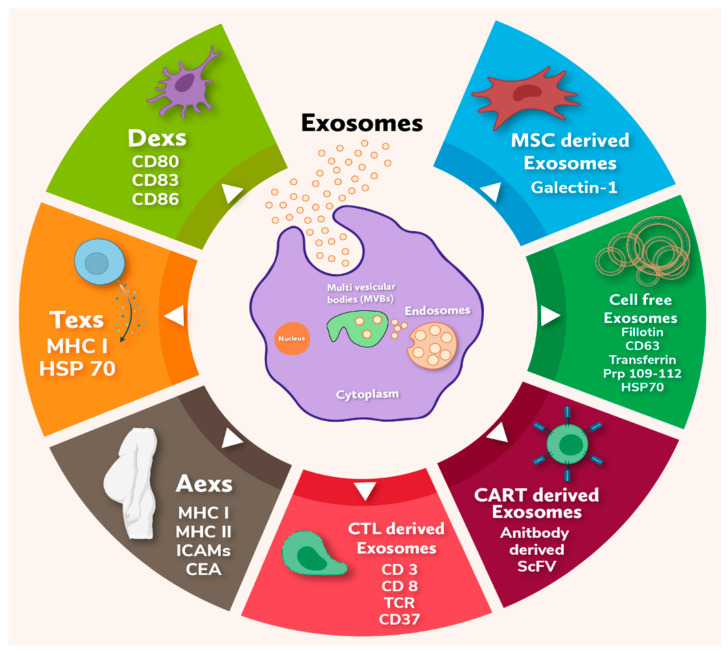
The diagrammatic representation depicts the release of different types of exosomes from the cells and their molecular markers (Icons are created with biorender.com (accessed on 20 June 2021)).

There are several types of exosomes depending on their site of origin: DCs-derived exosomes, Tumor-derived exosomes, Ascites-derived exosomes (Aexs), CTL derived exosomes, CAR-T (Cytotoxic T Lymphocyte) cells-derived exosomes, Mesenchymal stem cell-derived exosomes (MSCs) and natural source derived exosomes which are discussed below in detail and represented in Figure 2.

### 3.1. DCs-Derived Exosomes (Dexs)

Dendritic cells (DCs), involved in the first stage of cancer immunity, aims to activate tumor-specific cytotoxic lymphocytes, leading to the destruction of tumor cells [48]. The first FDA-approved DC vaccine to be used as immunotherapy for castration-resistant prostate cancer showed an average survival of 4.1 months (25.8 months in the ciprofloxacin-T group and 21.7 months in the placebo group) [49]. However, the DC vaccine consists of living cells, making it really expensive in terms of storage and stability over a longer period of time. Dexs carry numerous DC molecules associated with immune function including peptide/Major Histocompatibility Complex (MHC) complexes that trigger the response of antigen-specific T lymphocytes [50,51], and co-stimulatory molecules, in particular CD80, CD83, CD86, which further aid in the enhancement and initiation of T lymphocyte cells. Exposing adenocarcinoma cells to Dex treatment also causes an increase in the induction of interferon secretion [52,53]. These studies suggest that Dex maintains an essential immunostimulatory power of DCs, which could become a promising tool for cancer immunotherapy in future.

### 3.2. Tumor-Derived Exosomes (Texs)

Texs carry MHC-I, HSP70 and antigens speculated to be the source of specific stimuli against immune response exerted by cancer cells. Texs elicit an enhanced anti-tumor reaction more efficiently than the cancer cell debris, apoptotic materials and irradiated tumor cells [54]. HSP70, a stress-inducible exosomal heat shock protein that promotes NK cell activation and cancer cell lysis via granzyme B, acts as an endogenous danger signal to increase the immunogenicity of tumors by induction of CTL response [55]. Texs can also effectively release a variety of tumor antigens to DC; thus, they can be exploited as antigen carriers for cancer immunotherapeutics [56]. Texs are known to play a key role in cancer growth and progression, such as inducing apoptosis in activated CD8+ T cells, inhibiting immune cell proliferation, interfering with the monocyte differentiation, suppressing NK cell activity and encouraging Treg and MDSC expansion [57]. These effects come by directly suppressing the proliferation and inhibiting the cytotoxicity of NK cells or binding directly to the T-cells associated with HER2 receptors leading to the activation of multiple cells to inhibit tumor growth. In addition, the removal of PD-L1 leads to the anti-tumor properties, hence becoming as one of the potential therapeutic target [58]. Similar to the Dexs, Texs might also become a potential and immunogenic acellular vaccine [59].

### 3.3. Ascites-Derived Exosomes (Aexs)

Aexes are another form of exosome shown to play an important role in carcinogenesis. Aexs contain MHC-I and –II molecules, co-stimulatory molecules, ICAMs and the immunogenic carcinoembryonic antigen (CEA) which APCs may recognize. Initial clinical trials in advanced CRC patients have shown promising anti -tumor response of Aexs along with GM-CSF (Granulocyte-Macrophage Cell Simulating Factor) and may serve as alternative to immunotherapy [60]. 

### 3.4. CTLs Derived Exosomes

In the year 1989, Peters et al. suggested that exosomes derived from human T cells participate in the interaction of CTLs and the target cells [61]. However, in specificity towards CTLs, the presence of CD3, CD8 and TCR on CTLs derived exosomes could provide cytotoxicity to the targeted cells through TCR (T- Cell Receptor) interaction with the antigen/MHC-I complex. Such interaction may result in the target cell death [62], due to the presence of cytotoxic compounds in exosomes, including perforin, granzymes and lysosomal enzymes [63]. Early studies have emphasized that the accelerated secretion of exosomes by CTLs through TCR activation and TCR/CD3ζ complex has existed on the surface membrane of exosomes derived from human CTL [64], resulting in the rapid elimination of the target cell and thus serving and contributing to the adaptive immunity. 

### 3.5. Exosomes Derived from CAR-T Cells

CAR-T cell-derived exosomes may possess antibody-derived single-chain variable fragment (scFv), a promising alternative to cell therapy. Cellular communications between CAR-T or CTL and cancer cells are required for the anti-tumor effect of CAR-T cells and CTLs especially in an aggressive tumor. Both CAR-T cells and CTLs interaction with the cancer cells require the penetration of the CAR-T or CTLs cells in the tumor. However, the tumor milieu can limit the mode of action of CAR-T cells and CTLs as the scFv may influence the CAR-T cell function [65]. Consequently, this may limit clinical application of CAR-T-based cell therapy particularly in many solid tumors [66]. However, the adoptive transfer of CAR-T cells proposes an innovative method in cancer immunotherapy by provoking prompt and long-lasting clinical responses albeit with acute toxicities [67]. The exosomes released by CAR-T cells carry CAR on their surface, which helps in releasing highly cytotoxic molecules, thus inhibiting tumor growth. CAR exosomes do not express programmed cell death protein 1 (PD1) and, in contrast with CAR-T cells, their anti-tumor effect is uninfluenced by recombinant PD-L1 treatment. In addition, CAR exosomes have less toxicity and thus safer than CAR-T based cell therapy [63]. Having said that, CAR-T cell administered in vivo have shown significant tumor suppression and thus the use of CAR-T cell exosomes against triple negative breast cancer (TBNC) expressing MSLN may provide significant therapeutic benefit [68]. 

### 3.6. Mesenchymal Stem Cell-Derived Exosomes (MSCs)

MSCs are the important components in tissue repair/wound healing and can also produce exosomes at a very large scale [67]. MSC-exosomes also play a role in apoptosis of the activated T cells as they express galactin-1, a carbohydrate-binding protein that binds to the distinct set of glycoprotein receptors and acts extracellularly to induce cell death. MSCs can also pack mRNA into exosomes, preventing tumor migration and infiltration to distant areas. MSC-derived exosomes can also transmit extracellular miR-143 to osteosarcoma cells, which significantly decreases the migration of osteosarcoma cells. In addition, the discharge of MSC-derived exosomes miR-23B causes cell cycle suppression and dysfunction of breast cancer cells, thus preventing cancer cell migration and infiltration [69,70]. These exosomes stimulate the secretion of Interleukin-6 (IL-6), Interferon-γ (IFN-γ), Tumor necrosis factor- α (TNF-α) along with Activated B cells, T cells and Antigen presenting Cells (APCs) containing HoxB4. This affects the DC maturation and promotes T cell proliferation, differentiation and activation through the WNT signaling pathway [67]. These findings need to be further explored extensively for better therapeutics.

### 3.7. Exosomes Derived from Natural Sources

Interestingly, exosomes are also derived from plant sources and food/edible materials. Food derived exosomes (FDEs) are involved in the transport of biomolecules for cell-to-cell communication. These small vesicles (50–300 nm) are surrounded by a phospholipid bilayer and form intraluminal vesicles (ILVs) in multi vesicular bodies. These bodies fuse with the plasma membrane to produce ILVs in the extracellular environment and are referred to as exosomes [71,72]. Plant-derived exosome-like particles have gained much attention because of their source of origin and are known as Plant-derived edible nanoparticles (PDENs). They are found in the paramural space of plants and are identical in structure and function to their mammalian counterparts [73]. PDENs respond differently in different biological conditions; variation in the size and surface charge of exosomes depends on the plant source and environment. In the stomach and intestinal environment, grape-derived exosome-like vesicles reduced in size compared to vesicles suspended in water, while a fraction of ginger-derived vesicles expanded in stomach and intestine [74]. In addition, large number of exosome-like vesicles has been identified from ginger (Aprox. 50 mg per 1 kg of ginger) which are rich in proteins, lipids and other nuclear components [26]. The epidemiological studies suggest that continuous human exposure to exosomes of pasteurized milk may confer substantial risk for the development of chronic diseases including obesity, type 2 diabetes mellitus, osteoporosis, common cancers such as prostate, breast, liver, B-cells and Parkinson’s disease [26]. 

## 4. Exosomal Biomarkers in Breast Cancer

As the basic principles of exosome biology and their relationship with cancer and drug resistance are better understood, exosomes and the tumor microenvironment are increasingly becoming attractive targets for clinical applications; primarily due to their versatile role in carcinogenesis in terms of cancer diagnostic and treatment response [75]. Subsequently, exosomes can portray the entire tumor milieu because of their ability to be secreted from any cancer cell type [76]. It has been observed that exosome secretion has a direct relationship with the severity of cancer lesions, which may not only detect the disease but also the type of disease [76,77]. Circulating exosome-encapsulated miRNAs have been observed as ideal biomarkers for breast cancer for its good correlation with disease progression. For example, significantly high amount of exosomal miRNAs such as ci-miRNA-27 and ci-miRNA-365 are found in triple negative breast cancer patients compared to hormone receptor positive breast cancer patients [77,78,79,80]. Exosomes are shown to preserve miRNAs as cell-free miRNAs, as they are found in purified human peripheral blood micro-vessels. Subsequently, various studies show exosomal miRNAs in the blood as novel biomarkers for the diagnostic and prognostic evaluation of various human cancers including breast cancer [81,82,83,84,85,86,87]. In situ detection of miRNAs has highlighted that miR-21 could be a potential biomarker for both MCF-7 cells derived and normal cell-derived exosomes. In addition, miR-16 was also found to be transferred from murine breast cancer-derived TAMs via tumor-derived exosomes that prevent infiltration and polarization of macrophages in the tumor niche [88]. Exosomes derived from TAMs, containing miR-223 promote the invasive potential of breast cancer cells, thus promoting tumorigenesis [89]. Studies have also shown that the elevated level of TAMs resulting in a poor prognosis of breast cancer. However, TAM-derived exosomes might play a significant role in controlling disease progression and treatment via miRNA secretion. Consequently, exosomal miRNAs may critically impact breast cancer proliferation: metastasis, drug resistance, microenvironment formation and immune response. Some significant miRNAs are discussed in Table 1. Moreover, isolation of tumor markers in liquid biopsies is easy and cost-effective than solid tissue biopsies [90]. However, the physiognomies of circulating tumor cells (CTC) and cell-free DNA (cf-DNA) related to cancer are still unclear as compared to the exosomes of solid tumor biopsies. Furthermore, cf-DNAs carry mutations distinctively of the consistent primary tumors. In contrast, more circulating tumor DNA clearance is usually observed in the liver or kidneys, indicating steadiness and pathogenicity of circulating tumor DNA [91]. Exosomes containing different markers are represented in Table 2 and Figure 2.

## 5. Exosomes in Breast Cancer Aggressiveness

Communication of cancer cells with neighboring cells is crucial for tumor development, and it may happen through direct cell to cell or intracellularly with the help of some secretary molecules [126,127]. Exosomes produced from tumors are capable of promoting tumor cell proliferation and metastasis. Apart from their pro-tumorigenic activities, exosomes also contribute to tumor-tumor communication via chemoresistance transmission. Corcoran and colleagues first demonstrated that exosomes could convey Docetaxel resistance in prostate cancer [128], similar events have been observed in a variety of tumors such as hepatocellular, lung, liver including breast cancers [129,130,131]. Exosomes derived from tumors also interact with non-transformed differentiated cells, triggering the development of malignant characteristics in these target cells. For example, exosomes mediate intercellular communication between neoplastic and normal cells, resulting in the latter developing a pro-inflammatory phenotype. Exosomes from arsenite-treated liver cells were demonstrated to activate the IL6, IL8/STAT3 pathway, thereby increasing miR155 expression and inflammatory characteristics in normal liver cells [132].

In addition, tumor-derived exosomes play a critical role in tumor invasion by promoting tumor cell viability along with extracellular matrix degradation through matrix metalloproteinases (MMPs). They also exclude apoptosis-inducing proteins, specifically leading to the escape of tumor cells from immune surveillance [133,134]. HSP90+ exosomes derived from metastatic breast cancer cells and released exotically with the help of rab27b, can promote tumor invasion via degradation of extracellular matrix and activation of MMP2 [135]. Studies have also highlighted that exosome derived from linoleic acid-induced MDA-MB-231 can reduce E-cadherin expression while enhancing the expression of Snail 1/Snail 2, Twist 1/Twist 2, Vimentin, N-cadherin and Sip1 [136]. It has also been observed that exosomes derived from breast cancer cells contain miR-105, which regulates the tight junction protein ZO1 in recipient endothelial cells, may lead to augmented vascular permeability by downregulating the levels of ZOI [98]. Furthermore, recent research suggest that breast cancer-derived exosomes play a compelling role in organ-specific metastasis and angiogenesis as they contain annexin A2, which mediates brain and lung metastasis in particular [137]. An improved understanding of their mechanism may allow important therapeutic implications.

## 6. Exosomes as Drug Carriers

Exosomes have a low immune prototype, and thus have minor adverse effects [138]. Furthermore, exosomes can easily enter cells due to interactions between exosome membrane proteins and recipient cells [139], which makes them the most effective natural carrier for drug delivery. However, the origin of exosomes, techniques of purification, forms of drug loading and the final drug delivery system needs to be elucidated [17]. Tumor derived exosomes can deliver drugs more precisely to tumor cells and suppress tumor progression as seen in case of paclitaxel delivery to prostate cancer [140]. Similarly, exosomes from pancreatic cancer cells could effectively transfer curcumin to pancreatic cancer cells and cause considerable cell death [141]. In general, drug-loaded exosomes show better efficacy than chemical drugs alone. Furthermore, Kim et al. discovered that paclitaxel-loaded macrophage-derived exosomes had higher stability and loading efficiency than other drug-loading approaches, inhibiting Lewis Lung Carcinoma cell proliferation more effectively and showing anti-tumor activity in a murine Lewis Lung Carcinoma model [142]. In addition, Yong T et al. developed biocompatible tumor cell-exocytosed exosome-sheathed PSiNPs (E-PSiNPs) as a drug carrier for targeted cancer chemotherapy, which resulted in greater in vivo enrichment in total tumor cells and side population cells with CSC-like characteristics. The treatment also showed remarkable anticancer and CSC-killing activity in subcutaneous, orthotopic and metastatic tumors [143]. The administration of doxorubicin-loaded exosomes generated from DCs can significantly decrease breast tumor cell proliferation with no toxicity in mice. When DC-derived exosomes are combined with specific IRGD peptides, the exosomes have the ability to target breast cancer more effectively than a chemical formulation alone [144]. Exosomes containing cisplatin can prolong the life of ovarian cancer mice without generating liver or kidney side effects, which is an advantage over cisplatin alone. Additionally, exosomes containing cisplatin have an anti-tumor impact, in vivo and in vitro [145]. Curcumin loaded exosome of a murine lymphoma cell line may be successfully transferred to brain tissue, causing microglia death in the brain. These findings suggest that the strategy could provide a noninvasive and innovative therapeutic approach for treating brain inflammatory illnesses [146]. Mesenchymal stem cell-derived exosomes have been used to load miR-146b, resulting in effective inhibition of tumor growth [147,148]. These findings suggest that exosomes may be used as effective drug delivery vehicle with minimal side effects, however, more evidence are needed to use exosomes as drug delivery system.

## 7. Exosomes in Multidrug Resistance

Breast cancer exosomes can bind to selective therapeutic antibodies that can lead to treatment failure due to drug adsorption. Exosomes isolated from Her2+ breast cancer cell supernatants or serum can bind to trastuzumab, inhibiting its activity. The finding suggests that Her2+ exosomes may be used as a biomarker in trastuzumab-resistant tumor aggressiveness [118,149,150]. Various molecule such as transient receptor channel 5 (TrpC5), P-glycoprotein (P-gp), Survivin, DOX, mtDNA, Glutathione S-transferase P1 (GSTP1), ubiquitin carboxy-terminal hydrolase L1 (UCH-L1) etc. are linked with exosome mediated drug resistance [115,117,120,121,150,151,152]. Therefore, tumor derived exosome may not only serve as non-invasive biomarkers to explore the mechanism of drug resistance in breast cancer cases but also lead to personalized medicine or therapeutic interventions.

## 8. Exosomes in Breast Cancer Diagnosis

Recent research shows the presence of exosomes in nearly all body fluids, including blood, urine, saliva, breast milk, cerebrospinal fluid, semen, amniotic fluid and ascites [153]. Few studies have also proposed the utility of exosomes in the diagnosis and prognosis of different types of cancers. Particularly, in breast cancer, differential secretion of exosomes displaying an array of proteins such as Tetraspanin CD9, HSP70, Annexin-1 and metalloprotease ADAM10 at various stages of breast cancer may contribute to an accurate diagnosis and prognosis [114,150]. For example, tetraspanin CD63, an integrin-binding partner exclusively present on exosomes, expression correlates inversely with the cancer metastasis [154,155,156]. Del-1 and exosomal survival-2B (pro-apoptotic protein) can be used for differentiating benign/non-cancerous breast tumor [123] and a diagnostic and/or prognostic marker in patients with early breast cancer, respectively [119]. Along with several proteins, tumor-derived exosomal miRNAs such as miR16 also contribute to tumor evasion, leading to tumor progression. Mechanistically, exosomes derived from cancerous cells modifies the tumor microenvironment, which can eventually trigger immune cells to release epigallocatechin gallate (EGCG) [157]. Further mechanistic elucidation of proteins and miRNAs derived from circulating plasma exosomes can act as an early diagnostic, prognostic as well as therapeutic tool in cases of breast cancer.

## 9. Exosomes in Immune Response and Immunotherapy

Recent research findings indicate a distinct advantage of immunotherapy over existing conventional therapies [158]. Exosomes, derived from the cancer cells including breast cancer can modify the immune response by interacting with various immune cells, e.g., macrophages, regulatory T cells (Tregs), dendritic cells (DCs) and T cells [159]. Studies in breast cancer have also demonstrated that exosomal miRNAs transport stimulate the macrophages and contribute to angiogenesis [160]. Exosomes derived from murine breast cancer 4T1 cells took up fibronectin leading to an active interaction with immune cells when co-cultured with tumor infiltrating leukocytes [161]. The release of protein-coated exosomes called PD-L1, part of immune checkpoint protein family actively involved in immune surveillance, in melanoma skin cancer models and in blood samples of the people treated for breast and lung cancer [162] suggest a novel method to increase the efficacy of exosomes dependent tumor vaccines.

In addition, immunocyte exosomes include cytokines that govern inflammatory responses, innate immunity and lymphocyte production, among other processes. The research team of Gao, et al. found that Dex contains TNF-α, which could activate NF-KB by releasing membrane-bound TNF-α suggesting an involvement in endothelial inflammation and atherosclerosis [163]. Exosomes released by DCs, and macrophages include membrane-bound IL-1, which could be involved in inflammation [164,165]. Wang et al. found that the TGF-β-containing thymic cell-based exosomes boost T-cell development to Foxp3+ Tregs, the differentiation of CD4+CD25 T-cells from Tregs into the effector and their in vitro and in vivo proliferation [165]. Findings mentioned above suggest that exosomes may control key immunologic processes, release cytokines, regulate inflammatory response and innate immunity and also imply that immune cell exosomes may govern stem cell mobilization, tissue remodeling and immunological regulation.

## 10. Clinical Application of Exosomes

Recently, cancer cells secreted exosomes have become one of the emerging research areas in understanding cancer, especially breast carcinogenesis. Additionally, it also provides us an opportunity to explore biomarkers for better diagnosis and prognosis at an early stage [166,167]. In 2016, two test kits on fluid biopsy-based approaches were available to detect prostate and lung diseases (ExoDx^®^ prostate and ExoDx^®^ Lung, Exosome Diagnostics Inc., Waltham, MA, U.S.A) [87,168,169]. Breast cancer-derived exosomes have also been considered as a potential indicator of cancer progression [114]. However, further investigation is needed. Several proteins, including epidermal growth factor receptor (EGFR), survival apoptosis inhibitor, carcinogenic marker CD24, localized adhesive kinase (FAK) and surface cell proteoglycan glycan-1, are significantly overexpressed in the breast cancer patient’s serum-derived exosomes as compared to the healthy donors [119,170]. Researchers found a higher level of exosomes derived 27-hydroxycholesterol exosomes in MCF-7 when compared to MDA-MB-231 cells [171,172]. Exosomes derived epigallocatechin gallate (EGCG)-treated breast cancer cells when incubated with TAM in vivo, were found to repress M2 polarization and NF-κB signaling led to anti-tumor immune response [88]. Such studies indicate the potential use of exosomes as a promising agent for drug delivery vehicles in anti-tumor therapy. Furthermore, exosomes can both spread and curb the infections, and thus are considered as suitable candidates for developing vaccines for prevention and treatment [173]. The vaccine developed from exosomes was effective in anti-tumor immunity, however, further research is warranted to exploit its potency as a therapeutic candidate.

## 11. Future Prospects

Availability of limited therapies against breast cancer particularly TNBCs cause higher mortality than other subtypes among breast cancer patients. Ample evidence indicates the role of exosome and/or sEVs in carcinogenesis and thus can be used for diagnosis. Moreover, exosomes may act as a bridge for cellular communication in the tumor microenvironment resulting in tumor development, invasion, metastasis and drug resistance. Apart from their role in cancer progression, these could serve as a potential vehicle for inhibiting tumor growth and development by manipulating them for drug development and immune-surveillance. 

Recent path-breaking research tools such as immunotherapeutics (PDL-1, CAR-T, etc.) have immensely benefitted patients. Importantly, exosome-based immunotherapeutics exoPDL1, type of exosome-related immunotherapy, can be used to design drugs with minimum toxicity and greater clinical benefits. The small size of the exosomes makes them useful natural carriers for drug delivery into the cancer cell and may significantly contribute to therapeutic use. 

## 12. Conclusions

It is desirable that the ongoing efforts in cancer research should not only focus on the role of exosomes or sEVs in vitro but also on their significance in liquid biopsies, immunotherapy, drug designing and drug delivery systems to benefit patients greatly particularly in triple- negative breast cancer patients. 

## Figures and Tables

**Table 1:** List of some important exosomal miRNAs related to breast cancer.

S.No.	Description/Function	miRNAs Involved	Refs.
1	Exosomal miRNAs in breast cancer cell proliferation and apoptosis	miR-10a, miR-10b, miR-21, miR-27a, miR-155 and miR-373	[45]
		miR-21 and miR-10b	[92,93]
		miR-128	[94]
2	Exosomal miRNAs in breast cancer metastasis	miR-200a, miR-200b, miR-200c, miR-429 and miR-141	[95]
		miR-200c and miR-141	[95]
3	Exosomal miRNAs in drug sensitivity and resistance in breast cancer	miR-100, miR-17, miR-222, miR-342–3p and miR-451	[44]
		miR-4443, miR-574–3p, miR-7847–3p, miR-423–5p, miR-4298, miR-3178, miR-6780b-3p, miR-7107–5p, miR-744–5p, miR-4258, miR-138–5p and miR-210–3p	[96]
		miR-221/222	[97]
		miR-9	[98]
		miR-939	[99]
		miRNA-122	[100]
		miR-23b and miR-320b	[101]
4	Exosomal miRNAs in breast cancer tumor microenvironment	miR-21, miR-378e and miR-143	[102]
		miR-127, miR-197, miR-222 and miR-223	[103]
		MiR-503	[104]
		Exosomal miR-198, miR-26a, miR34a and miR-494	[19]
		miR-134	[105]
		miR-182	[106]
		miR-101 and miR-372	[107]
		miR-21 and miR-1246	[80]
		exosomal miR-1246	[108]
		miR-105	[98]
		miRNA-10b	[109]
		miR19a	[110]
		miR-338-3p, miR-340-5p and miR124-3p	[111]
		miR-29b-3p, miR-20b-5p, miR17-5p, miR-130a-3p, miR-18a-5p, miR-195-5p, miR-486-5p and miR-93-5p	[111]
		miR-221/222	[112]
		miRNA-451	[113]

**Table 2:** List of exosomal protein markers involved in breast cancer.

S.No.	Expression Site	Protein Markers	Refs.
1	Serum/pleural effusion-derived exosomes from breast cancer patients or cell lines	ADAM10, HSP70, CD9, Annexin1,	[114]
		TrpC5	[115]
		Glucose transporter 1 (GLUT-1), glypican 1 (GPC-1),	[116]
		Glutathione S-transferase P1(GSTP-1)	[117]
		HER-2	[118]
		Survivin (Survivn 2B)	[119]
		P-glycoprotein/TrpC5/ABCG2	[120]
		Ubiquitin carboxyl terminal hydrolase-L1 (UCH-L1)	[121]
		CD24, tetraspanins and epithelial cell adhesion molecule (EpCam)	[122]
2	Plasma	Developmental endothelial Locus-1 (Del-1) and fibronectin	[123,124]
		Fibronectin	[124]
4	Total blood	SERPINA1, KRT6B and SOCS3, IGF2R	[125]
